# Dermal Exposure Assessment to Pesticides in Farming Systems in Developing Countries: Comparison of Models 

**DOI:** 10.3390/ijerph120504670

**Published:** 2015-04-29

**Authors:** Camilo Lesmes Fabian, Claudia R. Binder

**Affiliations:** 1 Centro de Investigaciones de Ingenierias “Alberto Magno” (CIIAM); Universidad Santo Tomas Seccional Tunja; Sede Campus: Av. Universitaria Calle 48, No. 1-235 Este, 15001 Tunja, Boyaca, Colombia; 2 Department of Geography, Ludwig Maximilian University of Munich, Luisenstrasse 37, D-80333 Munich, Germany, E-Mail: claudia.binder@lmu.de

**Keywords:** dermal exposure assessment, modelling, pesticides, farming systems, potato crops, developing countries, Colombia

## Abstract

In the field of occupational hygiene, researchers have been working on developing appropriate methods to estimate human exposure to pesticides in order to assess the risk and therefore to take the due decisions to improve the pesticide management process and reduce the health risks. This paper evaluates dermal exposure models to find the most appropriate. Eight models (*i.e.*, COSHH, DERM, DREAM, EASE, PHED, RISKOFDERM, STOFFENMANAGER and PFAM) were evaluated according to a multi-criteria analysis and from these results five models (*i.e.*, DERM, DREAM, PHED, RISKOFDERM and PFAM) were selected for the assessment of dermal exposure in the case study of the potato farming system in the Andean highlands of Vereda La Hoya, Colombia. The results show that the models provide different dermal exposure estimations which are not comparable. However, because of the simplicity of the algorithm and the specificity of the determinants, the DERM, DREAM and PFAM models were found to be the most appropriate although their estimations might be more accurate if specific determinants are included for the case studies in developing countries.

## 1. Introduction

### 1.1. The Pesticide Issues

Pesticides are key elements of pest management programs in modern agriculture to increase the levels of production. Their use is stimulated by the commercialization and intensification of agriculture, the difficulty in expanding cropped acreage, the increased demand for agricultural products as the population increases, and the shift to cash crops for domestic and export sales [1]. It is estimated that annually some 2.5 million tons of pesticide are used worldwide and 220,000 people die because of poisoning from these substances. Most of these poisonings occur in developing countries because of weak safety standards, minimal use of protective equipment, absence of washing facilities, poor labeling, and lack of information programs [2,3,4,5,6]. 

Public health experts have expressed increasing concern about the use of pesticides because epidemiological studies have found that they are associated with different types of cancers [7,8,9,10], neurologic pathologies [11,12,13], respiratory symptoms [14] and hormonal and reproductive abnormalities [15,16,17,18,19]. Regardless of the risks involved in the use of pesticides, they are considered a key input to agriculture allowing intensive production techniques [20]. Therefore, it is crucial to assess the risk due to pesticide use by improving their management, reducing the exposure and protecting human health. 

The agricultural sector in Colombia uses 3.8 million hectares of land for permanent and transitory crops. During the last decade, an average of 82,000 tons of pesticides were applied per year (17% insecticides, 47% herbicides and 35% fungicides and bactericides) [21]. This suggests that part of the population and the environment in Colombia are likely to be exposed to negative effects derived from pesticide use. For instance, the potato farming system occupies 128,700 ha with 230,000 production units which had a production of 2.3 million tons in 2012 and used 32.5 kg/ha of pesticide active ingredients [22]. Therefore, the quantification of human exposure to pesticide use in farming systems like potato crops is crucial to provide information about the level of risk faced by farmers and workers and to support the development of proper policy measures.

### 1.2. Risk Assessment of Pesticide Use in Developing Countries

In the agricultural field, there is an increasing concern about the health of farmers, workers and bystanders, since they might be frequently exposed to pesticides for long periods of time. Governments, especially from developed countries, have introduced new environmental policies about the adequate use of pesticides. Meanwhile, in developing countries, like Colombia, a similar attempt has been done but even though the regulation scheme is already defined, this is not efficiently implemented due to the lack of information about exposure assessment and risk characterization [23,24]. The definition and implementation of these environmental policies is a further step after a risk assessment. Therefore, it is crucial to establish a method for the risk assessment of pesticide application in developing countries focusing in the exposure assessment and the risk characterization. The conclusions coming out from this method will be useful for stakeholders not only for the improvement of the risk assessment scheme, identifying the critical factors that influence the level of exposure concentrations, but also for the development of pedagogical programs about the appropriate use of pesticides. 

The risk assessment of pesticide application can be divided into two essential parts: *exposure assessment* (qualitative and quantitative description of the exposure concentrations and related dose for specific pathways) and *effects assessment* (determination of the intrinsic hazards associated with the agent and quantification of the relationship between the dose with the target tissue and related harmful outcomes) [25,26,27,28]. The first part is known as the initial portion of the environmental health paradigm: from sources, to environmental concentrations, to exposure, to dose. The effects assessment is aiming for the latter portion of the events continuum: from dose to adverse health effects. 

In the occupational hygiene field, the attention has shifted to the research of the exposure in the agricultural workplace to improve the pesticide management and to reduce the health risk [28]. This is of special interest in developing countries because pesticide management activities face weak safety standards [3,5,6,29]. Studies in potato farming systems in Vereda La Hoya, Colombia [3,5,23,24,30,31,32,33], Mojanda, Ecuador [34] and El Angel, Ecuador [35] have shown that pesticide management has no a particular theoretical basis and instead it is performed by trial and error finding out what works out in practice. Furthermore, farmers do not wear adequate personal protective equipment, apply pesticides which are banned in industrialized countries and modify the standard discharge of nozzles to reduce the application time [31]. Because these issues increase the health risk due to human exposure, a risk assessment of pesticide use in these areas is required in order to determine the risk level. 

### 1.3. Modeling Dermal Exposure to Pesticide Use 

Indirect methods to assess human exposure have been used since the early 1990s [36]. Tools for dermal exposure, such as Control of Substances Hazardous to Health (COSHH) regulations [37], Dermal Exposure Assessment Method (DREAM) [38], Estimation and Assessment of Substance Exposure (EASE) [39], European Predictive Operator Exposure Model Database (EUROPOEM) [40], Pesticides Handlers Exposure Database (PHED) [41], Risk Assessment of Occupational Dermal Exposure to Chemicals (RISKOFDERM) [42], Qualitative Assessment of Occupational Health Risks (STOFENMANAGER) [43], and the approaches proposed by the U.S. EPA [44] are targeted at occupational situations encountered in industrial processes in Europe and the USA, but they do not consider agricultural processes such as pesticide management and there might be uncertainties when they are applied in study areas in developing countries. Dermal Exposure Ranking Method (DERM) [45] is a method focused on occupational activities in pesticide management in developing countries; nonetheless, its semi-quantitative estimations still lack reliability and validity [46,47]. Pesticide Flow Analysis Model (PFAM) [48] is a model focused on farming systems in developing countries based on the material flow analysis method, however, it is still not validated. Because of the lack of studies about the application and further evaluation of these models in farming systems in developing countries, there is no consensus about the best method to evaluate dermal exposure and the health risk in those systems. Therefore, existing models for dermal exposure (DERM, DREAM, PHED, RISKOFDERM, COSHH, STOFENMANAGER, EASE and PFAM) were evaluated in order to find out the most appropriate to be applied in case studies in developing countries. Along this evaluation the following research questions were addressed:

1. Which of the existing models for dermal exposure assessment are feasible to be applied in case studies in farming systems in developing countries?
2. According to the parameters and determinants included in the model structure, which model assessment is more complete in terms of the evaluation of dermal exposure?
3. When comparing the model outcomes with the dermal exposure measurements in the study area, which model assesses dermal exposure more accurately?

## 2. Methodology

### 2.1. Multi-Criteria Analysis 

After a literature review, eight available models were considered for the analysis: COSHH [37], DERM [45], DREAM [38], EASE [39], PHED [41], PFAM [48], RISKOFDERM [42], and STOFENMANAGER [43]. These models were selected because of their availability, clear model description and their potential applicability for the assessment of pesticide use in farming systems in developing countries. They were analyzed according to a group of criteria such as availability, guidance, knowledge required, reliability, type of outcome, type of substance, target group, dermal exposure descriptor and dermal exposure pathway which are explained in Table 1.

**Table 1:** Description of the qualitative scoring system for the multi-criteria analysis.

Criteria	Qualitative Scoring		
	Low	Medium	High
*Target Group* (The model evaluation must be focused on farming systems)	Industry	Small and Medium Enterprises (SME)	Farms
*Guidance* (A guidance explaining the model evaluation is important for the model implementation)	No guidance available	Guidance on website	Guidance is published together with a paper
*Knowledge Required* (The model must be easy to apply on case studies in developing countries)	No special knowledge required	Basic computer and technical knowledge required	Advance computer knowledge required like programming and modelling
*Reliability* (The model is more reliable when it is already validated)	The model outcomes are not reliable according to the experts	The model outcomes are partly reliable as the model is partly validated	The model outcomes are reliable as the model is validated
*Outcome* (The dermal exposure assessment is more accurate when the models give a quantitative outcome)	The model outcome is qualitative	The model outcome is semi-quantitative	The model outcome is quantitative
*Evaluated Substances* (The model that includes a large amount and type of substances is a more adequate model)	Pesticides are not included in the assessment	Only Pesticides are included in the assessment	Pesticides and other chemicals are included in the assessment
*Dermal Exposure Descriptor* (The model must be focused on the actual exposure for a better risk assessment)	The model evaluates only the potential exposure	The model evaluates potential and actual exposure	The model evaluation is focused on the actual exposure
*Evaluated Body Parts* (Dermal exposure estimations are more accurate when the whole body is included in the assessment)	The model does not include any body parts in the assessment	Parts of the body are included in the model evaluation	The whole body is included in the model evaluation

### 2.2. Estimation of Dermal Exposures in the Study Areas 

From the results of the multi-criteria analysis and based on the model characteristics five models (*i.e*., DERM, DREAM, PFAM, PHED, and RISKOFDERM) were selected to be applied in the case study of potato farming systems in Vereda La Hoya in the highlands of Colombia. The data used as input comes from a previous survey made in the study area with 197 smallholder potato growers in four communities [3] and previous studies about dermal exposure in the same study area [24,31]. The input data and the scoring system for each determinant within each model are shown in the annexes. Because PFAM model required a specific pesticide with the total amount applied per hectare, the dermal exposure assessment was estimated for the pesticide methamidophos.

### 2.3. Description of the Study Area 

The study area is located in Vereda La Hoya near Tunja, the capital city of the province of Boyacá, Colombia. This is a rural region devoted mainly to the cultivation of potato in production units of around 3 hectares in size. The crop depends on rainfall, therefore, the production is generally organized into two periods, one from March to September and another from October to February, which corresponds to the two rainy seasons. Average annual productivity is 18.3 ton/ha [22]. Potato crops in this region are vulnerable to three major pests: the soil-dwelling larvae of the Andean weevil (*Premnotrypes vorax*), the late blight fungus (*Phytophthora infestans*) and the Guatemalan potato moth (*Tecia solanivora*) [22]. These pests, together with the weeds present in the early phases of the crop, are controlled by the application of chlorothalonil, chlorpyrifos, cymoxanil, glyphosate, mancozeb, methamidophos and paraquat [5,32]. In the study area the pesticide management is performed along three main activities: the preparation of the pesticide, the application itself, and the cleaning of the spraying equipment. During the whole pesticide management, farmers use work clothing consisting of trousers, short-sleeve shirts and plastic boots. These three activities consist of the following series of characteristics: (a) *Preparation:* This activity includes opening the bottle containing the pure pesticide substance, mixing the solution of (different) pesticides and water, and loading the tank of the knapsack sprayer. Farmers in Vereda La Hoya prepare the pesticides in a 100-L or 200-L capacity container. The pesticide and the water (normally 80 L to obtain four applications of 20 L each) are mixed in this container with the aid of a wooden stick. During the mixing and the filling of the tank there are usually spills out of the container affecting different parts of the body including hands, arms, chest and legs; (b) *Application:* Once the knapsack sprayer is carried on the back, the pesticide application starts with the spraying process on the field. During this activity the farmers’ body is exposed to the droplets emitted by the nozzles. In the study area the spraying is performed with hand pressure sprayers which are, on average, 9 years old [3,24]. They consist of a tank with a 20-L capacity, an injection and pressure system with an external piston pump and a pressure chamber with a capacity of 21 bar, a spraying pressure of 3 ± 0.3 bar and a pressure range between 1 and 14 bar. Farmers use two types of nozzles for pesticide application which differ in the amount of pesticide discharged: a high-discharge (HD) nozzle used during the first crop phases (sowing and emergence) and a low-discharge (LD) nozzle used during the rest of the crop phases (growth, flowering and pre-harvest). The discharges of the HD and LD nozzles measured in the study area were 1.88 ± 0.12 L/min (n = 24) measurements, and 1.26 ± 0.08 L/min (n = 24) respectively. Farmers purchase standard discharge nozzles of 1.05 ± 0.02 L/min (n = 8) and then modify the plastic and metal structures of the nozzles in order to obtain these discharges; (c) *Cleaning:* Once the application is finished, farmers clean the sprayer and the container by pouring clean water on all the accessories in a procedure repeated three times. This procedure is included in the booklet “Good Agricultural Practices” [49] which farmers use as a reference for the pesticide management. During this activity, there are numerous spills from the equipment and the accessories reaching the farmer’s body. Previous studies have measured the dermal exposure and made an attempt to assess the health risk. These results are shown in Table 2.

**Table 2:** Pesticides commonly used in Vereda La Hoya and their Health Risk Assessment [24,31].

Pesticide	Toxicity	Total Pesticide Applied (kg/ha·day)	Potential Dermal Exposure (mg/kg·day)	Actual Dermal Exposure (mg/kg·day)	Health Risk Assessment
Chlorothalonil	- Low acute toxicity - Probable carcinogen	0.54	47–70	2–3	Low
Chlorpyrifos	- Moderately toxic - Affect the nervous system	0.44	38–43	1–3	Moderate
Cymoxanil	- Slightly toxic; - Reproduction and development effects; - Eye irritant	0.08	7–11	0.3–0.4	Moderate
Glyphosate	- Slightly toxic; - Eye and skin irritant	0.14	12–18	0.6–0.7	Moderate
Mancozeb	- Slightly Toxic; - Carcinogen; - Reproduction and development effects; - Respiratory tract irritant	0.66	58–64	2–4	Moderate
Methamidophos	- Very toxic; - Mutagen; - Cholinesterase inhibitor; - Neurotoxicant	0.55	48–72	2–3	Very High
Paraquat	- Mutagen; - Respiratory tract irritant; - Eye irritant	0.08	7–11	0.3–0.4	Very High

## 3. Results and Discussion

### 3.1. Multi-Criteria Analysis 

**Figure 1:**
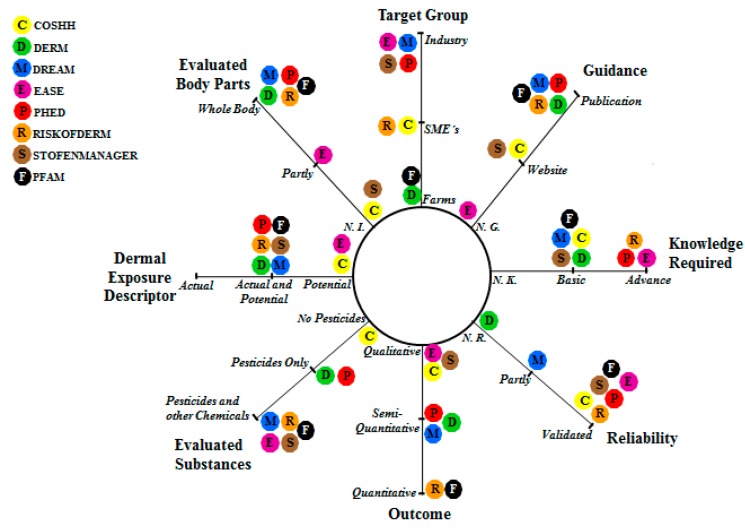
Radar diagram with the multi-criteria analysis for the evaluated models for dermal exposure assessment (NI: Not Included; NG: No Guidance; NK: No Knowledge Required; NR: Not Reliable).

The multi-criteria analysis found that only DERM, DREAM, PHED, RISKOFDERM and PFAM can feasibly be applied in case studies in developing countries (Figure 1, Table 3). COSHH was excluded from the evaluation as it does not consider important criteria relevant for case studies in developing countries such as target group, as it is focused on guidance for small and medium enterprises (SMEs), as it is only available in a website with a user’s manual for only some specific industries; concerning outcome, its assessment is qualitative; regarding evaluated substances, it does not evaluate pesticides in farming systems; its dermal exposure descriptor only assesses the potential exposure; and concerning evaluated body parts, it does make a distinction between any body part. EASE was also excluded from the evaluation as it does not consider criteria such as target group, it is focused on industrialized processes, for guidance there is no user’s manual with the model description; it provides a qualitative, its dermal exposure descriptor only evaluates the potential exposure and as to evaluated body parts, it only considers arms and forearms. STOFENMANAGER was also excluded from the evaluation as it does not comply with criteria such as target group, it is focused on industrial processes, the website does not show the algorithms or model calculations for guidance, its outcome assessment is qualitative and there is no information available regarding evaluated body parts. 

**Table 3:** Description of the evaluated models for dermal exposure assessment according to the multi-criteria analysis.

CRITERIA	MODELS							
	COSHH	DERM	DREAM	EASE	PHED	RISKOF	STOFFEN	PFAM
**Origin**	UK	Nicaragua	The Netherlands	UK	USA/Canada	Europe	The Netherlands	Switzerland
**Year**	2002	2008	2003	1994	2002	2003	2003	2013
**Goal**	Risk assessment in SMEs	Risk assessment in developing countries	Risk assessment of occupational exposure in any situation	Risk assessment for regulatory of new chemicals	Standardized exposure estimates	Risk assessment for regulatory and registration processes	Risk assessment in SMEs	Risk Assessment in developing countries
**Basis**	Operational exposure levels assess exposure and R-phrases for health hazard	Transport Processes, Schneider, 1999 [50]; DREAM, 2003 [38]	Transport processes, Schneider, 1999 [50]. Airborne concentrations [51]	Computer aided decision tree format [52], Schneider, 1999 [50]	Reported information on pesticides and monitoring data	Schneider, 1999 [50]; COSHH [37].	Schneider, 1999 [50]; COSHH [37]. Riskofderm [53]	Material Flow Analysis Methodology
**Target group**	SME’s	Farmers in developing countries	Industrial processes and farming systems	Industrial processes	Regulatory agencies, pesticide industry	Operational and technical staff mostly in SMEs	Dutch companies	Farming Systems in Developing Countries
**Availability**	Electronic version	Publication	Publication	Software available	Software and publication	Software and publication	Website	Publication
**Guidance**	Website with guidelines for specific industries	Publication	Publication	Not available	Publication	Publication	Website with no guidelines about the algorithms	Publication
**Knowledge/Equipment required**	No specific expertise required and electronic version available	Basic mathematics skills and easy to carry out in the field	Basic mathematics skills and easy to carry out in the field	Knowledge of the model and programming	Knowledge of the criteria and their effects on exposure. Computer required	Knowledge of the model and computer required	Internet access required	Basic mathematics skills
**Reliability**	Evaluated by the U.S National Institute for Occupational Safety and Health (NIOSH)	Not validated	Good inter-observer agreement	Distributed over 200 users in EU, USA, ASIA and Australia	Evaluated and approved by EPA	Developed by 15 European institutes based on a large database.	Widely used in The Netherlands	Good agreement with the dispersion scheme but still not validated
**Outcome**	Semi-quantitative (bands)	Semi-quantitative	Semi-quantitative	Quantifies the degree of exposure	Semi-quantitative	Quantitative	Ranking of risks in bands	Quantitative
**Type of evaluated substances**	Chemical products except pesticides	Pesticides	Metals, fluids and pesticides	Pure substances, no mixtures	Pesticides	Pure substances including pesticides	Pure substances and mixtures	Pesticides and other substances
**Evaluated dermal exposure pathway**	Deposition, indirect and direct contact	Transfer, deposition and emission	Transfer, deposition and emission	Emission to surface, air, outer clothing layers and direct to skin	No Data	Deposition and direct contact	Total dermal exposure	Transfer, deposition and emission
**Dermal exposure descriptor**	Potential exposure	Potential and actual exposure	Potential and actual exposure	Potential exposure	Potential and actual exposure	Potential and actual exposure	Potential and actual exposure	Potential and actual exposure
**Evaluated Body Parts**	No information available	Front and back side of neck, thorax, arms, forearms, hands, thighs, legs, feet, forehead and left and right side of face	Head, upper and lower arms, hands, front torso, back, upper legs, lower legs and feet	Hands and forearms	Head, face, back and front neck, chest/stomach, back, upper arms, forearms, hands, thighs, lower legs, feet.	Exposure is evaluated according to percentage of body exposed	No information available	Arms, forearmes, chest, abdomen, back, legs, thighs and hands.
**Reference**	[37]	[45]	[38]	[39]	[41]	[42]	[43]	[48]

### 3.2. Estimation of Dermal Exposures in the Study Areas 

According to the previous results DERM, DREAM, PHED, RISKOFDERM, and PFAM were selected as the most appropriate models to be applied in the case study of Vereda La Hoya. The determinants included in each model are shown in Table 4 and the input data consider for each model is given in the Appendix Table A1, Table A2, Table A3, Table A4 and Table A5. Even though the evaluated dermal exposure models provide insights into the level of exposure, their outcomes differ because of the model structure and the determinants included in each model structure (Table 5). Previous direct measurements in Vereda La Hoya found that dermal exposure to pesticides is very high (Table 2) because of the inadequate work clothing, the modification of nozzles to increase the discharge, the inappropriate cleaning of the application equipment, the pesticide application against the wind direction and the use of pesticide with a high level of toxicity [24,31].

Actual dermal exposure values were also found higher than the reference values for human exposure for some pesticides like metamidophos [24,31]. Therefore, from the comparison of the models estimations and the type of determinants considered by each model, DERM, DREAM, and PFAM were found to be the most appropriate models. However, PHED might give an inaccurate estimation because the model determinants are relevant for farming systems in industrialized countries. Even though the model includes pesticide application scenarios which might be useful for developing countries, the model does not assess processes like pesticide emission and transfer, important processes within the mass transport quantification which should be included in the conceptual model for dermal exposure assessment, according to Schneider [50]. RISKOFDERM estimation might also be inaccurate because the model evaluated the exposure according to a percentage of body exposed and the quantitative estimation cannot be compared with reference values of human exposure as the pesticides have different levels of toxicity and the model only gives a qualitative assessment of “high” based on the quantitative estimation. 

DERM is an appropriate model because of the specificity of the determinants for case studies in developing countries; however, the estimation accuracy might be underestimated because important determinants are not consider such as washing the equipment, task duration, wearing gloves, frequency and replacement of gloves, work clothing, personal hygiene and climate conditions. Therefore, this model has the potential to increase the accuracy of its estimations when these determinants are included in the assessment. DREAM was found to be an appropriate model as its estimation corroborates the dermal exposure assessment made in the location [24,31]; however, the estimation accuracy might be improved if there is a differentiation in the protection factor according to the different body parts and other determinants are considered such as climate conditions like wind speed and humidity. If these missing determinants are included the model scope will be wider for not only farming systems in industrialized and developing countries but other industrial processes. Finally, PFAM was found to give a quantitative assessment in terms of potential and actual exposure and how the protection factor influences the actual exposure. In addition it can assess the risk for each pesticide separately. However, it needs to be calibrated with direct measurements before it can be implemented in study areas with the same characteristics. Nevertheless, this model has the advantage of complying with all the required criteria in order to be implemented in case studies in developing countries. 

These results are valid for potato farming systems and many other crop systems with similar characteristics in different regions in Latin America and might be also be valid for other regions worldwide with similar pesticide applications in Africa or Asia. However, the results are not valid for other sophisticated pesticide applications in crops in developing countries such as flowers, banana, coffee, sugar cane, rice, *etc.*

All the models for human exposure such as COSHH [37], DREAM [38], EASE [39], PHED [41], RISKOFDERM [42] and STOFENMANAGER [43] were developed after the conceptual model proposed by Schneider in 1999 [50,51]. Therefore, they were developed with similarities in the structure of the determinants. However, they are built for case studies in industrialized countries and there are uncertainties about their application in developing countries. For instance COSHH is specialized in SMEs in the UK; DREAM, in industrialized countries and farming systems in The Netherlands where tractors and motorized pesticide applications are used; EASE, in industrialized processes in the UK; PHED, in regulatory agencies and the pesticide industry in the USA and Canada; RISKOFDERM, for operational and technical staff in SMEs; and, STOFFENMANAGER, for Dutch companies. Some agricultural case studies in developing countries are characterized by manual pesticide applications with no regulations about the adequate pesticide use and no use of personal protection equipment. Only the DREAM model was applied in study areas in developing countries but the model has not been validated because of some issues regarding the reproducibility and accuracy of dermal exposure estimations [54]. Furthermore, this research found that when this model is applied in case studies in developing countries, most of the determinants do not cover the specific characteristics of these study areas. Based on DREAM, Blanco attempted to develop a model for farming systems in developing countries with DERM; however, this model has faced problems in the validation because of inappropriate procedures in the methodology [47]. However, despite this inaccuracies in the estimations of all the evaluated models, their structure has the potential to redefine and include other determinants which might be the origin to create a brand new model for dermal and human exposure assessment in farming systems in the developing world. 

## 4. Conclusions 

This research evaluated models for dermal exposure assessment focusing on case studies in developing countries. From the multi-criteria analysis and the type of determinants included in the models, DERM, DREAM, PHED, PFAM and RISKOFDERM were found as the most appropriate models to assess the dermal exposure in developing countries. Regarding the specificity to the farming systems in developing countries, DERM, DREAM and PFAM include determinants which are relevant for the system characteristics in the study area. However, all the five selected models are suitable to be modified in their structure in order to include parameters or determinants which might increase the accuracy of the estimations. 

**Table 4:** Determinants considered by the Evaluated Models.

DERM	DREAM	PHED	RISKOFDERM	PFAM
- Sprayed surface - Height of the crop - Leaking backpack - Volume of sprayed dilution - Nozzle height - Spraying in front - Spraying against wind - Splash/spill over the pump - Splashes on hands - Splashes on feet - Gross contamination of the handsa. Wearing long sleeved shirtb Wearing short sleeved shirt - Wearing an old/overused/torn shirta Wearing long pantsb Wearing short pants - Wearing old/overused/torn pants - Wearing shoes	- Emission to clothing and uncovered skin; and immersion of skin into agent - Intensity of emission - Exposure route factors (emission, deposition, transfer) - Probability of deposition on clothing and uncovered skin - Intensity of deposition on clothing and uncovered skin - Transfer to clothing and uncovered skin - Intensity of transfer - Body surface factor - Physical state - Concentration - Evaporation (liquids): Boiling temperature - Viscosity - Formulation - Dusty (solids) - Stickiness/wax/ moist (non-powder/ non-dusty solids) - Glove or clothing material - Protection factor - Replacement frequency - If non-woven gloves connect well to clothing of arms - If non-woven gloves are worn during total time of task - A second pair of gloves is worn under outer gloves - Replacement frequency of these inner gloves - Barrier cream used - Relative task durationa. Categorical estimateb Absolute estimate - Worker’s hygiene factor - Continued exposure - Hygiene estimate work Environment	- Mixing status - Using enclosed mixing system - Application method - Tractor with enclosed cab/charcoal filter - Repair status - Washing equipment - PPE use - Replacing gloves - Personal Hygiene - Change clothes after a spill	- Route weight fraction - Substance specific modifier - Workplace modifier - Control measure modifier - Default exposure values by task group - Clothing protection factor - Activity time - Exposed body area	- Pesticide preparation - Pesticide application - Pesticide cleaning - Potential exposure - Protection Factor-Actual Exposure - Total Exposure

**Table 5:** Actual dermal exposure assessments by the selected models for the study area.

Model	Model Scoring Ranges		Unit	Scores for the Case Study by the Evaluated Models	Qualitative Assessment by the Evaluated Models
	Lowest Value	Highest Value			
DERM	0	>150	Unitless	44.28	Moderate
DREAM	0	>1000	Unitless	359.0	Very High
PHED	0.05	>30	Unitless	15.2	High
PFAM	0	∞	mg/kg.day	2.36–2.71	Very High
RISKOFDERM	0	>30	mg/cm²/h	0.65	High

The evaluated models have the possibility to assess industrial and agricultural processes in industrialized and developing countries. However, DREAM was found to have a number and type of determinants that not only increase the accuracy of the estimation but they might serve as a basis to develop a new model including more determinants with higher specificity to study areas in farming systems in developing countries.

Previous studies found that because of the inadequate work clothing, the modification of nozzles to increase the discharge, the inappropriate cleaning of the application equipment, the pesticide application against the wind direction and the use of pesticides with a high level of toxicity, the dermal exposure was assessed as very high because both the potential and actual exposure for some pesticides were higher than the reference values for human exposure. Therefore, when comparing these results with the model estimations, it was found that DREAM and PFAM gave the most accurate estimations. However, it is important to take into account that DREAM is a semi-quantitative model easy to apply in the case studies. On the contrary, PFAM gives a quantitative estimation but the transfer coefficients must be determined in the field in order to calibrate the model. 

## Appendix

**Table A1:** DERM Scoring System for the Case Study.

Nr.	Name	DERM Scoring	System Characteristics	Scores for the Case Study
1	Sprayed surface	(a) ≤ 0.7 ha = 1 (b) > 0.7 ha = 2	According to the survey made in the study area, the average size of the crop field is 0.98 ± 0.75 ha	(b) >0.7 ha = 2
2	Height of the crop	(a) 1 × 1 = 1 (b) 1 × 2 = 2 (c) 1 × 3 = 3 (d) 1 × 4 = 4 (e) 1 × 5 = 5 (f) 3 × 1 = 3 (g) 3 × 2 = 6 (h) 3 × 3 = 9 (i) 3 × 4 = 12 (j) 3 × 5 = 15	The first number means: (1) Previously contaminated surfaces; (3) Recently contaminated surfaces. The numbers 1 to 5 represent the percentage ranges of the total body surface (0–20, 21–40, 41–60, 61–80, 81–100). Because the potato crops grow up to 60 cm, the values are: 3 for recently contaminated surfaces and 2 for 40% of the body exposed.	(g) 3 × 2 = 6
3	Leaking backpack	(a) 0 (b) 5 × 1 = 5 (c) 5 × 2 = 10 (d) 5 × 3 = 15 (e) 5 × 4 = 20 (f) 5 × 5 = 25	There is evidence that during the whole pesticide application procedure, there is a leaking in the sprayer and the upper back is exposed.	(b) 5 × 1 = 5
4	Volume of sprayed dilution	(a) ≤30 liters = 2,5 (b) >30 liters = 5	Because of the extension of the crop fields, normally the amount of sprayed dilution is approximately 20 L.	(a) 2,5
5	Nozzle height	(a) 4 × 1 = 4 (b) 4 × 2 = 8 (c) 4 × 3 = 12 (d) 4 × 4 = 16 (e) 4 × 5 = 25	The nozzle height has a potential exposure of 60% of the body.	(c) 4 × 3 = 12
6	Spraying in front	(a) 0 ((b) 5 × 1 = 5 (c) 5 × 2 = 10 (d) 5 × 3 = 15 (e) 5 × 4 = 20 (f) 5 × 5 = 25	There is a potential exposure in 60% of the body surface.	(d) 5 × 3 = 15
7	Spraying against wind	(a) 0 ((b) 5 × 1 = 5 (c) 5 × 2 = 10 (d) 5 × 3 = 15 (e) 5 × 4 = 20 (f) 5 × 5 = 25	There is a potential exposure in 60% of the body surface as the region has a strong wind.	(d) 5 × 3 = 15
8	Splash/spill over the pump	(a) 0 (b) 1 × 1 = 1 (c) 1 × 2 = 2 (d) 1 × 3 = 3 (e) 1 × 4 = 4 (f) 1 × 5 = 5 (g) 3 × 1 = 3 (h) 3 × 2 = 6 (i) 3 × 3 = 9 (j) 3 × 4 = 12 (k) 3 × 5 = 15	The potential exposure is limited to hands and arms	(d) 3 × 1 = 3
9	Splashes on hands	(a) 0 (b) 5 × 1 = 5 (c) 5 × 2 = 10 (d) 5 × 3 = 15 (e) 5 × 4 = 20 (f) 5 × 5 = 25	The potential exposure is limited to hands	(b) 5 × 1 = 5
10	Splashes on feet	(a) 0 (b) 5 × 1 = 5 (c) 5 × 2 = 10 (d) 5 × 3 = 15 (e) 5 × 4 = 20 (f) 5 × 5 = 25	The potential exposure is limited to feet	(b) 5 × 1 = 5
11	Gross contamination of the hands	(a) 0 (b) 5 × 1 = 5 (c) 5 × 2 = 10 (d) 5 × 3 = 15 (e) 5 × 4 = 20 (f) 5 × 5 = 25	Gross contamination of hands occur by blocking a hose leakage, repairing nozzle or mixing the pesticide	(b) 5 × 1 = 5
12	a. Wearing long sleeved shirtb. Wearing short sleeved shirt	(a) 0 a. (b) 0.20 b. (c) 0.15	The clothing protection is assumed 0 when there is no protection and 0.15 for short sleeve shirts and 0.20 for long sleeve shirts. Farmers use short sleeve shirts	(c) 0,15
13	Wearing an old/overused/torn shirt	(a) 0	Farmers always apply the pesticides with overused/old or torn shirts	(a) 0
14	a. Wearing long pants b. Wearing short pants	(a) 0 a. ((b) 0.15 b. ((c) 0.10	In general farmers wear trousers with thicker fabrics in long pants	(b) 0.15
15	Wearing old/overused/torn pants	(a) 0	Farmers always apply the pesticides with overused/old or torn pants	(a) 0
16	Wearing shoes	(a) 0 (b) 0.10	Farmers protect the feet with boots.	(b) 0.10

**Table A2:** DREAM Scoring System for the Case Study.

Nr.	Name	DREAM Scoring	System Characteristics	Scores for the Case Study
1	Emission to clothing and uncovered skin; and immersion of skin into agent (P_E.BP_)	(a) <1% of task duration = 0 (b) <10% of task duration = 1 (c) 10–50% of task duration = 3 (d) ≥50% of task duration = 10	There is a potential emission during the whole process of the pesticide application.	(d) ≥50% of task duration = 10
2	Intensity of emission (I*E.BP*)	(a) <10% of body part = 1 (b) 10–50% of body part = 3 (c) ≥50% of body part = 10	There is evidence that more than 50% of the body surface is exposed	(b) ≥50% of body part = 10
3	Exposure route factors (emission, deposition, transfer)(ER_E_, ER_D_, ER_T_*)*	(a) Emission = 3 (b) Deposition = 1 (c) Transfer = 1	The system covers these three processes.	(a) Emission = 3 (b) Deposition = 1 (c) Transfer = 1
4	Probability of deposition on clothing and uncoverd skin (P_D_._BP_)	(a) <1% of task duration = 0 (b) <10% of task duration = 1 (c) 10–50% of task duration = 3 (d) ≥50% of task duration = 10	There is a pesticide deposition on the clothing and uncovered skin during the whole pesticide application.	(d) ≥50% of task duration = 10
5	Intensity of deposition on clothing and uncovered skin (I*_D.BP_*)	(a) <10 % of body part = 1 (b) 10–50% of body part = 3 (c) ≥50% of body part = 10	The deposition on clothing covers more than 50% of the body surface	(b) 10–50% of body part = 3
6	Transfer to clothing and uncovered skin (P*_T.BP_*)	(a) <1% of task duration = 0 (b) <10% of task duration = 1 (c) 10–50% of task duration = 3 (d) ≥50% of task duration = 10	There is a transfer to clothing and uncovered skin during some of the pesticide management activities.	(c) 10–50% of task duration = 3
7	Intensity of transfer (I*T.BP*)	(a) not contaminated = 0 (b) possibly contamination = 1 (c) <50% of contact surface = 3 (d) ≥50% of contact surface =10	There is a high intensity of transfer	(b) <50% of contact surface = 3
8	Body surface factor (BS_BP_)	(a) Head (BS_HE) = 0.69 (b) Upper arm (BS_U(A) = 0.67 (c) Forearm (BS_F(A) = 0.53 (d) Hands (BS_H(A) = 0.47 (e) Torso front (BS_TF) = 1.22 (f) Torso back (BS_T(B) = 1.22 (g) Lower body part (BS_L(B) = 2.43 (h) Lower leg (BS_LL) = 1.15 (i) Feet (BS_FE) = 0.63	This factor is given by the model	(a) Head (BS_HE) = 0.69 (b) Upper arm (BS_U(A) = 0.67 (c) Forearm (BS_F(A) = 0.53 (d) Hands (BS_H(A) = 0.47 (e) Torso front (BS_TF) = 1.22 (f) Torso back (BS_T(B) = 1.22 (g) Lower body part (BS_L(B) = 2.43 (h) Lower leg (BS_LL) = 1.15 (i) Feet (BS_FE) = 0.63
9	Physical state (PS)	(a) Solid = 1 (b) Liquid = 1 (c) Vapour-gaseous = 0.3	Pesticides are applied in a dilution.	(b) Liquid = 1
10	Concentration ((C)	(a) >90% active ingredient of interest = 1 (b) 1–90% active ingredient of interest = 0.3 (b) (c) <1% active ingredient of interest = 0.1	The pesticides are usually diluted	(b) 1–90% active ingredient of interest = 0.3
11	Evaporation (liquids): Boiling temperature (EV)	(a) <50 °C = 3 (b) 50–150 °C = 1 (c) >150 °C = 0.3	Pesticides are always diluted, therefore the value 1 was considered	(b) 50–150 °C = 1
12	Viscosity (V)	(a) Low, like water = 1 (b) Medium, like oil = 1.75 (c) High, like resin/paste = 3	Because of pesticides dilutions, the viscosity was considered as 1, like water.	(a) Low, like water = 1
13	Formulation (F)	(a) fine particles (powder) = 3 (b) granules/grain/pellets = 1 (c) pack/bunch/bundle = 0.3	Some of the pesticides are available as fine particles in order to be diluted in water.	(a) fine particles (powder) = 3
14	Dusty (solids) (DU)	(a) No = 1 (b) Yes = 3	While mixing, dust can occur.	(b) Yes = 3
15	Stickiness/wax/ moist (non-powder/non-dusty solids) (SS)	(a) No = 1 (b) Yes = 1.75	Water was used to dilute the chemicals.	(a) No = 1
16	Glove or clothing material (M)	(a) No gloves/clothing used = 1 (b) Woven clothing = 0.3 (c) Non-woven permeable = 0.1 (d) Non-woven impermeable = 0.03	Normally farmers use gloves in some activities and woven clothing material.	(b) Woven clothing = 0.3
17	Protection factor (PFM_HA_/PFM_BP_)	(a) PFMHA = 1 (b) PFMBP = 0.3	Farmers use work clothing and gloves	(a) PFMHA = 1 (b) PFMBP = 0.3
18	Replacement frequency (RF)	(a) Used once = 0.3 (b) Daily = 1 (c) Weekly = 3 (d) Monthly = 10	The work clothing is used weekly	(c) Weekly = 3
19	If non-woven gloves connect well to clothing of arms (G(C)	(a) No = 3 (b) Yes = 1	The farmers do not use non-woven gloves.
20	If non-woven gloves are worn during total time of task (G(D)	(a) 0–25% of task duration = 10 (b) 25–99% of task duration = 3 (c) 100% of task duration = 1	The farmers do not use non-woven gloves.
21	A second pair of gloves is worn under outer gloves (UG)	(a) No = 1 (b) Yes = 0.3	There is no use of a second pair of gloves under the outer gloves.	(a) No = 1
22	Replacement frequency of these inner gloves (URF)	(a) After 1 time = 1 (b) Daily = 3 (c) ≥Weely = 10	No inner gloves were used.
23	Barrier cream used (B(C)	(a) No = 1 (b) Yes = 0.3	Farmers in the study area do not use barrier cream.	(a) No = 1
24	Relative task duration (RT(D)a. Categorical estimate (CAT)b. Absolute estimate (ABS)	(a) Daily 4–8 h/weekly >20 h/monthly >80 h/yearly >800 h = 1 (b) Daily 1–4 h/weekly 4–20 h/monthly 16–80 h/yearly 160–800 h = 0.3 (c) Daily 11–60 min/weekly 1–4 h/monthly 4–16 h/yearly 40–160 h = 0.1 (d) Daily <11 min/weekly 0–1 h/monthly 0–4 h/yearly 0–40 h = 0.03 (a) Total time of task performance/total working time	The total working time in which there is a potential dermal exposure is 5 hours	a. (a) Daily 4–8 h/weekly >20 h/monthly >80 h/yearly >800 h = 1
25–26	Worker's hygiene factor (WH)	(a) Hands not washed = 1 (b) Washed 2–10 times per shift with water = 0.3 (c) Washed 2–5 times per shift (scru(b) soap/solvents = 0.3 (d) Washed >10 times per shift with water = 0.1 (e) Washed >5 times per shift with (scru(b) soap/solvents = 0.1	There are two moments in which farmers wash their hands: before 1 break and before lunch	(b) Washed 2–10 times per shift with water = 0.3
27–29	Continued exposure (CE)	(a) Working clothes are immediately changed after work: No = 0.3, Yes = 1 (b) Workers responsible for washing own working clothes: No = 1, Yes = 3 (c) Workers immediately shower after work: No = 1, Yes = 0.3	Farmers change their clothes after the working time	(a) Working clothes are immediately changed after work: Yes = 1 (b) Workers responsible for washing own working clothes: No = 0.3 (c) Workers immediately shower after work: Yes = 1
30–33	Hygiene estimate work environment (EH)	(a) Daily cleaning wet = 0,1 (b) Weekly cleaning wet = 0.3 (c) Cleaning dry = 1	In general, after the application of pesticides the farmer cleans the equipment by rinsing it with clean water.	(a) Daily cleaning wet = 0.1

**Table A3:** PHED Scoring System for the Case Study.

Nr.	Name	PHED Scoring	System Characteristics	Scores for the Case Study
1	Mixing status	(a) Never = 0 (b) <50% of time mixed = 3 (c) >50% of time mixed = 9		The pesticide solution is mixed with different chemicals in water.	(c) >50% of time mixed = 9
2	Using enclosed mixing system	(a) Yes = 0.5 (b) (b) No = 1.0		Pesticides are mixed in 80–200 L container and in the field.	(b) No = 1.0
3	Application method	(a) Doesn’t apply = 0 For herbicides (b) Aerial-aircraft = 1 (c) Distribute tablets = 1 (d) In furrow/banded = 2 (e) Boom on tractor = 3 (f) Backpack = 8 (g) Hand spray = 9 For crop insecticides (h) Aerial-aircraft = 1 (i) Seed treatment = 1 (j) Distribute tablets = 1 (k) In furrow/banded = 2 (l) Boom on tractor = 3 (m) Backpack = 8 (n) Hand spray = 9 (o) Airblast = 9 (p) Mist blower/ fogger = 9	For animal insecticides (q) Ear tags = 1 (r) Inject animal = 2 (s) Dip animal = 5 (t) Spray animal = 6 (u) Pour on animal = 7 (v) Powder duster = 9 For fungicides (w) Seed treatment = 1 (x) Distribute tablets = 1 (y) In furrow/banded = 2 (z) Boom on tractor = 3 (aa) Backpack = 8 (ab) Hand spray = 9 (ac) Airblast = 9 (ad) Mist blower/fogger= 9 For fumigants (ae) Gas canister = 2 (af) Row fumigation = 4 (ag) Pour fumigant = 9	In the study area 96% of the farmers sprayed their pesticides (insecticides, fungicides, herbicides) with a backpack sprayer.	For herbicides (f) Backpack = 8 For crop insecticides (m) Backpack = 8 For fungicides (aa) Backpack = 8
4	Tractor with enclosed cab/charcoal filter	Boom, in furrow, hand spray, mist blower, airblast on tractor (a) Cab = Yes, Filter = Yes → = 0.1 (b) Cab = Yes, Filter = No → = 0.5 (c) Cab = No, or do not use tractor → = 1.0		In the study area tractors are not used.	(c) Cab = No, or do not use tractor → = 1.0
5	Repair status	(a) Doesn’t repair = 0 (b) Repair = 2		The sprayers used in in the study area are between 8 and 11 years old. Therefore multiple repairments are made.	(b) Repair = 2
6	Washing equipment	(a) Do not wash = 0 (b) Hose down sprayer = 0.5 (c) Hose down tractor = 0.5 (d) Clean nozzle = 3 (e) Rinse tank = 1		Farmers clean the equipment with water after the pesticide application.	(d) Clean nozzle = 3
7	PPE use	Scoring for Protection (a) PPE-0 = 1.0. Never used PPE (b) PPE-1 = 0.8. 20% Protection: One or more indicated PPE: Dusk mask, Full face shields, goggles, fabric/leather gloves, cloth overall (c) PPE-2 = 0.7. 30% Protection: Cartridge respirator, gas mask, chemical resistant boots, disposable outer clothing (Tyvek) (d) PPE-3 = 0.6. 40% Protection: chemical resistant rubber gloves (e) PPE-1 & PPE-2 = 0.5 (f) PPE-1 & PPE-3 = 0.4 (g) PPE-2 & PPE-3 = 0.3 (h) PPE-1 & PPE-2 & PPE-3 = 0.1		Farmers use the minimal protection like gloves and work clothing.	(b) PPE-1 = 0.8
8	Replacing gloves	Fabric/leather gloves (a) Change after each use = 1 (b) Change once a month or 1–4 times per person = 1.1 (c) Change when they are worn out = 1.2		Gloves are used until they are worn out.	(c) Change when they are worn out = 1.2
9	Personal Hygiene	(a) Hyg-1 (80% protection) = 0.2 (b) Hyg-2 (60% protection) = 0.4 (c) Hyg-3 (40% protection) = 0.6 (d) Hyg-4 (20% protection) = 0.8 (e) Hyg-5 (no protection) = 1.0		Farmers use a minimal protection and they have also minimal hygiene habits. However, these are not enough.	(c) Hyg-3 (40% protection) = 0.6
10	Change clothes after a spill	(a) Right away = 1.0 (b) Always use disposable clothing = 1.0 (c) At lunch = 1.1 (d) At the end of the day = 1.2 (e) At the end of the next day = 1.4 (f) Later in the week = 1.8		In the pesticide management, farmers use to clean change the clothes at the end of the day.	(d) At the end of the day = 1.2

**Table A4:** RISKOFDERM Scoring System for the Case Study.

Nr.	Name	RISKOFDERM Scoring	System Characteristics	Scores for the Case Study
1	Route weight fraction (RWF)	Hand tool dispersion: Body Hand (a) Direct contact (DC): 20% 30% (b) surface contact (SC): 50% 50% (c) deposition (DEP): 30% 30%	This DEO unit was best fitting our task group	Hand tool dispersion: Body Hand (a) Direct contact (DC): 20% 30% (b) surface contact (SC): 50% 50% (c) deposition (DEP): 30% 30%
2	Substance specific modifier	Volatility: Like water (DC 1, SC 1, DEP 1)	This data set was best fitting our task group	Volatility: Like water (DC 1, SC 1, DEP 1)
3	Workplace modifier	Spraying of liquids: Little pressure (DC 1, SC 0.3, DEP 0.1)	This data set was best fitting our task group	Spraying of liquids: Little pressure (DC 1, SC 0.3, DEP 0.1)
4	Control measure modifier	Level of automation: No automation (DC 1, SC 1, DEP 1)	This data set was best fitting our task group	Level of automation: No automation (DC 1, SC 1, DEP 1)
5	Default exposure values by task group	Spray dispersion of liquids: 0.459 (Body), 1.067 (Hand)	This default exposure value was best fitting our task group.	Spray dispersion of liquids: 0,459 (Body), 1,067 (Hand)
6	Clothing protection factor (CPF)	(a) light clothing = 0.5 (b) thick clothing = 0.1	The type of clothing depends on the clima of the day, both are possible.	(b) thick clothing = 0.1
7	Activity time (AT)	(a) <0.1 h = 0.1 (b) 0.1–0.5 h = 0.1 (c) 0.5–1 h = 0.3 (d) 1–4 h = 1 (e) >4 h = 3	The activity time of the farmers was between 1–4 h.	(e) >4 h = 3
8	Exposed body area (EBA)	(a) <10 (size of a large coin; small splashes) = 0.1 (b) 10–500 (one hand or less) = 0.3 (c) 501–2000 (hands and lower arms, or hands and head) = 1 (d) >2001 (more than hands and head) = 3	The exposed body area is assumed to be from very small to very high. It depends on the way the farmer works on the field.	(d) >2001 (more than hands and head) = 3

**Table A5:** PFAM Scoring System for the Case Study.

Nr.	Name	PFAM Scoring	System Characteristics	Scores for the Case Study
1	Amount of Applied Pesticide		The evaluations considered the application of 550 g of metamidophos per ha.	0.55 kg
2	Exposure during pesticide Preparation	Transfer Coefficient: 5.47E-5	Transfer coefficient considered when there are splits and splashes during the pesticide mixing.	Transfer Coefficient: 5.47E-5
3	Potential Exposure during pesticide Application	Transfer Coefficients: (a) Application with HD (High Discharge) Nozzles: 8.91E-4 (b) Application with LD (Low Discharge) Nozzles: 1.15E-3 (c) Application with SD (Standard Discharge) Nozzles: 7.72E-4	Farmers modify the nozzles and the two types of nozzles were considered.	(a) Application with HD (High Discharge) Nozzles: 8.91E-4 (b) Application with LD (Low Discharge) Nozzles: 1.15E-3
4	Protection factor	Transfer Coefficients: (a) Protection in the low body parts: (>90%) (b) Protection in the arms (HD: 51%, LD: 88%) (c) Protection in the upper back (HD: 74%, LD: 82%)	The protection factor given by work clothing and calculated for the application activity is high for legs, thighs, chest, abdomen and lower back (>90%) when both types of nozzles (HD and LD) are used. The protection factor is low in the arms (ranging from 51.8 to 88%) and also in the upper back (ranging from 74.8 to 82.6%).	(a) Protection in the low body parts: (>90%) (b) Protection in the arms (HD: 51%, LD: 88%) (c) Protection in the upper back (HD: 74%, LD: 82%)
5	Actual dermal exposure	Transfer Coefficients: (a) Application with HD Nozzles: 3.29E-5 (b) Application with LD Nozzles: 4.23E-5	Actual exposure depends on the protection factor and the potential exposure	(a) Application with HD Nozzles: 3.29E-5 (b) Application with LD Nozzles: 4.23E-5
