# Correction: Dermal Exposure Assessment to Pesticides in Farming Systems in Developing Countries: Comparison of Models. *Int. J. Environ. Res. Public Health* 2015*, 12,* 4670–4696 

**DOI:** 10.3390/ijerph120809264

**Published:** 2015-08-07

**Authors:** Camilo Lesmes-Fabian

**Affiliations:** Centro de Investigaciones de Ingenierias “Alberto Magno” (CIIAM), Universidad Santo Tomas Seccional Tunja, Sede Campus, Av. Universitaria Calle 48, No. 1-235 Este, Codigo 15001, Tunja, Boyaca, Colombia; E-Mail: camilo.lesmes@usantoto.edu.co; Tel.: +57-319-315-4121

We wish to make the following changes to the published article [1], agreed upon by all authors. 

Claudia R. Binder has withdrawn her co-authorship. The corrected author list should therefore read: Camilo Lesmes-Fabian.

Camilo Lesmes-Fabian thanks Claudia R. Binder for her comments and inputs to earlier drafts of this publication. 

The author would like to apologize for any inconvenience caused to readers by these changes.
